# The sigma-1 receptor as a neurohomeostatic decision hub for GABARAP-mediated receptor trafficking and macroautophagy

**DOI:** 10.3389/fmolb.2025.1673249

**Published:** 2025-10-30

**Authors:** Marius Wilhelm Baeken, Fazilet Bekbulat, Hagen Körschgen, Albrecht Martin Clement, Christian Behl

**Affiliations:** Institute of Pathobiochemistry, The Autophagy Lab, University Medical Center of the Johannes Gutenberg University Mainz, Mainz, Germany

**Keywords:** sigma-1 receptor, GABARAP, autophagy, GABAa receptor, LIR

## Abstract

Gamma-aminobutyric acid receptor-associated protein (GABARAP) is a multifunctional member of the autophagy-related (ATG8) protein family, playing key roles in two distinct cellular pathways: macroautophagy and plasma membrane protein trafficking. In the context of autophagy, GABARAP modulates cargo recognition and supports the maturation and fusion of autophagosomes with lysosomes, a critical step in intracellular clearance and proteostasis. Separately, GABARAP also regulates vesicular receptor protein transport from the Golgi apparatus to the plasma membrane, contributing to proper surface localization and receptor recycling. Both tasks are especially vital for neurons, where protein turnover and receptor localization are tightly linked to synaptic plasticity and neuroprotection. We recently identified a direct interaction between GABARAP and the sigma-1 receptor (σ_1_R), an ER-resident receptor involved in diverse cellular stress responses, mitochondrial function, and protein homeostasis. Our findings suggest that σ_1_R acts as an upstream regulatory hub, influencing GABARAP’s functional commitment to either membrane trafficking or autophagy. Specifically, we hypothesize that ligand-dependent σ_1_R activation promotes GABARAP’s involvement in macroautophagy at the expense of its role in membrane transport. This regulatory switch may underline part of the neuroprotective effects observed with σ_1_R agonists in neurodegenerative disease models, where enhanced autophagy is often beneficial. Overall, we discuss the emerging molecular crosstalk between σ_1_R and GABARAP, its potential impact on neuronal homeostasis, and how σ_1_R’s pharmacological modulation might be leveraged to bias GABARAP function toward autophagy in diseases such as amyotrophic lateral sclerosis, Huntington’s, Parkinson’s, and Alzheimer’s disease.

## Graphical Abstract

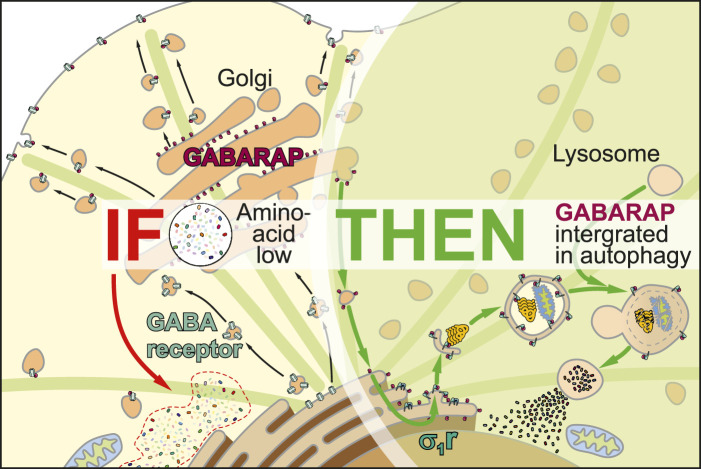

## Introduction

Post-mitotic cells, especially neurons, have to co-orchestrate numerous delicate homeostatic systems, such as protein or ion homeostasis, to ensure organismal function. Hence, a cross-talk between these systems is essential.

We recently described the sigma-1 receptor (σ_1_R), a protein lacking any close homologs in the human proteome (Moebius et al., 1997), as a regulator that integrates gamma-aminobutyric acid receptor-associated protein (GABARAP) into macroautophagy (Baeken et al., 2025; Christ et al., 2019). In brief, σ_1_R-deficient HeLa cells were unable to incorporate GABARAP into autophagosomes, as evidenced by a complete loss of GABARAP-p62 co-localization and a breakdown of the GABARAP-associated autophagic flux (Baeken et al., 2025). However, we noticed that GABARAP’s lipidated form still persisted, which suggests an interaction with membranous structures. Interestingly, a previous study has shown that the G^116^A (amino acids in single letter code) GABARAP mutant, which cannot be C-terminally cleaved, was unable to transport GABA_A_ receptors to the plasma membrane (Chen et al., 2007). This cleavage is a prerequisite for lipidation, which suggests that lipidation also facilitates the transport of associated vesicles.

Although still circumstantial, this evidence may broaden our understanding of σ_1_R’s physiological impact. As its *bona fide* cellular purpose remains elusive, we propose a regulatory role of σ_1_R in controlling GABARAP logistics, which affects neurotransmission as well as autophagy. In a broader context, this regulatory function might help explain the neuroprotective properties attributed to σ_1_R (Christ et al., 2020).

## GABARAP’s role in mammalian macroautophagy

Macroautophagy (hereafter autophagy) is a highly conserved cellular degradation pathway, characterized by the formation of double-membraned vesicles – autophagosomes – that engulf cytosolic material and fuse with lysosomes, where contents are degraded by hydrolases and recycled for reuse (He and Klionsky, 2009). Dynamically regulated, autophagy enables cells to deal with changing demands, be it upholding energy homeostasis during nutrient deprivation or selectively removing misfolded proteins and damaged organelles. Thus, autophagy is an essential homeostatic and adaptive process promoting survival and renovation, especially for post-mitotic cells such as neurons (Mizushima and Komatsu, 2011). Autophagic efficiency declines with age and its dysfunction is associated with disease, including neurodegeneration (Aman et al., 2021; Palmer et al., 2025).

Autophagosome formation, maturation, and fusion with lysosomes are coordinated by core autophagy-related (ATG) proteins, including an initiation complex, a membrane expansion complex, and two ubiquitin-like conjugation systems, one being the LC3/GABARAP system (Ariosa and Klionsky, 2016). Microtubule-associated protein 1A/1B-light chain 3 (LC3) and GABARAP, both members of the ATG8-family, form two distinct subfamilies: LC3A, LC3B, and LC3C; and GABARAP, GABARAPL1, and GABARAPL2 (Schaaf et al., 2016).

ATG8 proteins are ubiquitously expressed, though GABARAPs are enriched in the central nervous system and endocrine glands (Lee and Lee, 2016). Although highly homologous, distinct residues mediate selectivity of GABARAP subfamily members towards interaction partners (Sugawara et al., 2004; Rogov et al., 2014); emerging evidence supports unique and complementary roles for LC3s and GABARAPs in autophagy. During autophagosome formation, LC3B is mainly involved in the elongation of the phagophore, general cargo recognition, and the transport of autophagosomes to lysosomes, while GABARAP has primarily been associated with late events such as autophagosome closure and autophagosome-lysosome fusion (Lee and Lee, 2016; Schaaf et al., 2016). Supporting its function in autophagosome maturation, GABARAP shows a higher affinity to Unc-51 like autophagy activating kinase 1 (ULK1) (Alemu et al., 2012), and mediates autophagosomal targeting of phosphatidylinositol 4-kinase type 2 alpha (PI4KIIα), thereby regulating autophagosome-lysosome fusion (Albanesi et al., 2015).

To function in autophagy, cytosolic ATG8s must undergo lipidation, i.e., a covalent conjugation to phosphatidylethanolamine (PE), enabling incorporation into autophagosomal membranes (Lee and Lee, 2016; Nguyen et al., 2016; Palmer et al., 2025). Initially synthesized as precursors, ATG8s are cleaved by cysteine endopeptidases of the ATG4 family, whereby a C-terminal glycine residue becomes accessible for lipidation (Nguyen et al., 2020). The exposed glycine is then adenylated, sequentially transferred through E1-like and E2-like ATG proteins, and finally conjugated to PE’s amino head group via an E3-like ATG conjugation complex (Maruyama and Noda, 2017; Martens and Fracchiolla, 2020).

One hallmark of autophagosome-mediated autophagy is cargo-specific sequestration, in which adaptor proteins bind distinct ubiquitinated cargo and directly interact with inner autophagosomal membrane-bound ATG8 proteins (Khaminets et al., 2016), enabling selective elimination of various cellular components. The latter interaction is assured by distinct binding motifs termed LC3-interacting regions (LIRs), which are recognized by LIR docking sites in ATG8s (Johansen and Lamark, 2020).

The core of the canonical LIR is defined by the hydrophobic consensus sequence [W/F/Y]-X_1_-X_2_-[L/V/I] (X for variable residue) (Birgisdottir et al., 2013). Its affinity is enhanced by phosphorylated or acidic residues flanking the motif, albeit preferentially on the N-terminus (Johansen and Lamark, 2020). The ubiquitin-binding protein p62, considered a *bona fide* cargo adaptor, directly binds to all ATG8s (Pankiv et al., 2007; Johansen and Lamark, 2020). Specificity of cargo adaptors toward particular ATG8s arises from unique hydrophobic contacts, salt bridges, and hydrogen bonds. Notably, selectivity towards GABARAP is mediated by a restriction of this core motif: [W/F]-[V/I]-X-V (Rogov et al., 2017; Rogov et al., 2023).

## GABARAP’s role in receptor transport and deployment

**FIGURE 1:**
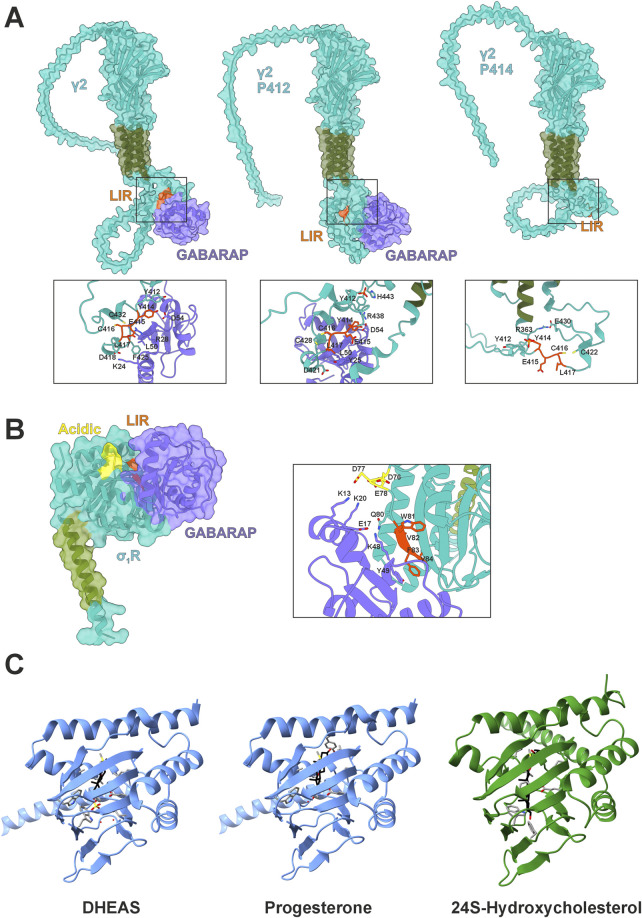
GABARAP interactions and σ_1_R structural context. **(A)** AlphaFold3 models of GABARAP (purple) in complex with the GABAA receptor subunit γ2 (teal with green transmembrane domain) with highlighted LIR (orange) in their native states including phosphorylated tyrosines Y^412^/Y^414^. **(B)** AlphaFold3 model of GABARAP (purple) in complex with σ_1_R (teal with green transmembrane domain), highlighting the LIR domain (orange) and acidic residues (yellow). **(C)** Crystal structure of *Xenopus laevis* σ_1_R (blue) with DHEAS (pdb:8WUE) or progesterone (pdb:8W4C) bound and Boltz-2 predicted interaction of *Homo sapiens* σ_1_R (green) with 24S-hydroxycholesterol.

Although widely known for its integration into autophagic processes, GABARAP was originally identified as a cellular agent to properly distribute GABA_A_ receptors to the plasma membrane by interacting with the γ2 subunit (GABRG2) of the heteropentamer (Wang et al., 1999). Following studies confirmed that GABARAP interacts with γ1, but not with the γ3 or δ subunits in yeast hybrid systems (Kneussel et al., 2000). The primary interaction site of GABARAP with GABA_A_ points to an intracellular loop between transmembrane regions 3 and 4 within the γ1/γ2 subunits (Nymann-Andersen et al., 2002; Chen et al., 2000). This interaction is facilitated by a classical LIR motif (^414^YECL^417^) (Ye et al., 2021). This core motif is also present in γ3 but lacks the favored acidic N-terminal residues (Rogov et al., 2017). Interestingly, Y^412^ in γ2 is phosphorylated by FYN and SRC kinases (Jurd et al., 2010), presumably regulating its affinity (Kittler et al., 2008). While Y^414^ can also be phosphorylated, the consequence for GABARAP binding remains unclear (Jurd et al., 2010). However, our *in silico* model suggests that this modification might affect binding negatively, whereas unmodified Y^414^ or pY^412^ would bind GABARAP (Figure 1A). Here, pY^412^ could enable intramolecular interaction with H^443^, condensing the C-terminal structure of γ2 and potentially stabilizing GABARAP binding.

Furthermore, GABARAP is involved in maintaining membrane GABA_A_ localization by several mechanisms. (i) GABARAP can negatively regulate GABA_A_ receptor reinternalization. This process relies on binding of the adaptor protein complex 2 (AP2) to the γ2 subunit (Comenencia-Ortiz et al., 2014). Coincidentally, GABARAP and AP2 compete for the same binding sites (Ye et al., 2021; Kittler et al., 2008). (ii) GABARAP partakes in GABA_A_ receptor recycling by interacting with endosomal receptors, thus rerouting them toward the plasma membrane (Lüscher and Keller, 2001). (iii) GABARAP interacts with gephyrin and ankyrin 3, which facilitate postsynaptic receptor clusters and GABA_A_ receptor stability (Nelson et al., 2020; Chen and Olsen, 2007). Moreover, lipidated GABARAP could potentially serve as an additional anchor for GABA_A_ receptors via its PE-tail.

GABARAP-mediated protein trafficking extends beyond GABA_A_ receptors; it is also involved in depositing angiotensin II receptor type 1 (AGTR1) at the plasma membrane (Cook et al., 2008). Mutagenesis analyses identified F^309^, L^316^, and L^317^ as critical residues, characterizing the motif as a canonical LIR (^313^FLQL^316^). This sequence, however, is not flanked by acidic but basic residues, defying the paradigm (Rogov et al., 2017). Notably, AGTR1 is highly expressed in dopaminergic neurons of the *substantia nigra pars compacta* that are highly susceptible to Parkinson’s disease (Kamath et al., 2022). Furthermore, GABARAP is responsible for the installation of transient receptor potential vanilloid 1 (TRPV1) at the plasma membrane (Laínez et al., 2010), which is important for microglial function in Alzheimer's disease (AD) (Lu et al., 2021). Here, the cytosolic ^791^FALV^794^ sequence might serve as a LIR. GABARAP also couples receptor homeostasis and autophagy by regulating epidermal growth factor receptor degradation via its LIR motif ^1086^FLPV^1089^ (Dobner et al., 2020; Üffing et al., 2024).

Dormant GABARAP localizes mainly to the Golgi, where it binds golgin subfamily A member 2 (GOLGA2) (Joachim et al., 2015), and to the ER, suggesting a role at the intersection of autophagy and receptor transport (Nakamura et al., 2008). Upon amino acid starvation, uncleaved GABARAP is displaced from GOLGA2 and rerouted from the Golgi to the ER via the centrosome (Joachim et al., 2015). How GABARAP is ultimately integrated into autophagy, and how these pathways are coordinated, remains unclear, though recent findings suggest a role for σ_1_R (Baeken et al., 2025).

## Structure-function relation of σ_1_R

As a modulatory receptor pre-dominantly localized at the mitochondrial associated membrane (MAM) (Aishwarya et al., 2021), the σ_1_R is involved in multiple cell physiological processes such as calcium signaling, NMDA receptor activity, G-protein-mediated signaling pathways, and autophagy (Couly et al., 2023). It thus contributes to neuronal excitability, plasticity, proteostasis, and resilience. Accordingly, σ_1_R agonists have been proposed as therapeutic candidates for neurodegenerative diseases (Couly et al., 2024; Lachance et al., 2023). First identified over four decades ago as a unique opioid receptor class due to its atypical ligand binding properties (Martin et al., 1976; Tam, 1983), the σ_1_R represents an evolutionary orphan, sharing no close homology with any other human protein.

Phylogenetically, σ_1_R shares ∼69% structural similarity with yeast sterol isomerases that are involved in ergosterol biosynthesis (Hanner et al., 1996). It is highly conserved among vertebrates, with 80% sequence identity to zebrafish (*Danio rerio*) (Schmidt et al., 2016). X-ray crystallography of purified σ_1_R revealed a homotrimeric organization, where each protomer contains a cupin-like β-barrel ligand-binding domain (LBD) flanked by four α-helices and a deeply buried ligand-binding site (Schmidt et al., 2016). Membrane anchoring likely occurs via a single, poorly conserved N-terminal transmembrane helix linked to E^102^ of the LBD by V^36^ and F^37^. The protein’s topology remains debated - type II (C-terminus in ER-lumen) versus type III (C-terminus cytosolic) – likely due to artifacts from protein tagging (Aydar et al., 2002; Hayashi and Su, 2007). Most structural studies support a type II orientation, placing the C-terminal LBD in the ER-lumen, consistent with its interaction with the luminal chaperone heat shock 70 kDa protein 5 (HSPA5) (Hayashi and Su, 2007; Mavylutov et al., 2018; Sharma et al., 2021). However, this poses questions about the function of the N-terminal di-basic ER retention motif (^1^MQWAVGRR^8^) (Seth et al., 1998; Aishwarya et al., 2021). Notably, since most experiments to date have employed tagged proteins, the *in situ* topology of endogenous σ_1_R remains unresolved.

While no specific signal transduction pathway has been defined for σ_1_R, evidence suggests that agonists promote its monomeric, active form, facilitating interactions with client proteins, while antagonists stabilize an inactive oligomeric state. Agonist binding leads to dissociation from HSPA5, allowing σ_1_R to stabilize inositol trisphosphate receptor (IP3R) at the MAM, consequently enhance Ca^2+^ efflux, or modulate NMDA receptor activity (Hayashi and Su, 2007; Balasuriya et al., 2013; Choi et al., 2017). Prolonged activation further drives σ_1_R translocation to nuclear and plasma membranes, expanding its functional repertoire (Tsai et al., 2015).

Agonist treatment features a biphasic dose response, suggesting that high concentrations may suppress σ_1_R activity (Maurice, 2021), this response varies by cell and agonist type and dosage. Overall, σ_1_R activation promotes the unfolded protein response (Alam et al., 2017) and autophagy (Christ et al., 2019; Yang et al., 2019; Wang et al., 2021), reducing ER stress and supporting proteostasis. Several mutations in σ_1_R disrupt this function and are linked to motoneuron diseases; E^102^Q, for instance, is implicated in juvenile-onset ALS (Al-Saif et al., 2011; Vollrath et al., 2014; Dreser et al., 2017) and F^83^L in an ALS-like condition (Ma et al., 2020). The latter mutation resides in a sequence we have recently identified as a LIR motif, ^81^WVFV^84^ with specificity for GABARAP (Figure 1B) (Baeken et al., 2025). AlphaFold modeling supports experimental data by predicting π-stacking between F^83^ of σ_1_R and Y^49^ of GABARAP, potentially explaining binding specificity despite F^83^’s variable motif position (Figure 1B). Notably, the agonist-mediated induction of σ_1_R’s monomeric state enables this interaction; we did not observe any interaction in an oligomeric state. This specific interaction between σ_1_R and GABARAP may provide a causal link for the well-established effects of σ_1_R on autophagy (Christ et al., 2019; Yang et al., 2019; Wang et al., 2023).

## σ_1_R as pharmaceutical target

The discovery that agonistic compounds such as alazocine [(+)-SKF-10047] and (+)-pentazocine (Martin et al., 1976; Su, 1982) selectively bind σ_1_R sparked its cellular and molecular characterization while highlighting it as a potent druggable target. It’s versatile roles as a regulator of Ca^2+^-homeostasis at the MAM, as a modulator of transmembrane receptors, and as an enhancer of the unfolded protein response and autophagy underline the potential neuroprotective properties of σ_1_R activation. Over time, extensive preclinical evidence has demonstrated that σ_1_R agonists confer neuroprotection across a broad spectrum of acute and chronic neurological disorders, including nerve injury, ALS, AD, Parkinson’s, and Huntington’s disease (Drewes et al., 2025).

Notably, neuroprotective σ_1_R agonists are structurally diverse. Classic benzomorphan-based ligands such as (+)-pentazocine (Li et al., 2021) and (+)-SKF-10047 (Phan et al., 2005), as well as chemically distinct molecules such as blarcamesine (Anavex 2-73), PRE-084 (Lahmy et al., 2013), and pridopidine (Ryskamp et al., 2019; Estévez-Silva et al., 2022), have all demonstrated beneficial behavioral and neuroprotective effects in various pathogenic mouse models. Several of these compounds have progressed to clinical trials. Pridopidine is currently in phase III for Huntington’s disease (NCT04556656) and phase II for ALS (NCT04615923); however, the HEALEY-ALS platform trial reported no significant clinical improvement (Shefner et al., 2025). In contrast, a phase IIB/III trial of blarcamesine in early AD has demonstrated significant improvements across multiple clinical outcomes (Macfarlane et al., 2025).

At the molecular level, σ_1_R agonists exhibit distinct modes of action, likely due to subtle differences at the agonist-σ_1_R interface, which may explain divergent cellular responses. Recent findings show that benzomorphan-based compounds – but not phencyclidine-derived compounds such as PRE-084 – induce monomerization of endogenous, untagged σ_1_R *in vitro* and in mouse tissues (Couly et al., 2024), at least under the experimental conditions and compound concentrations used. Moreover, the nature of the cellular stressor influences this response: oxidative stress (H_2_O_2_), but not ER stress (thapsigargin), promotes σ_1_R monomerization, even though both stressors activate the receptor (Couly et al., 2024). These results suggest that structurally diverse agonists and endogenous ligands regulate the switch between the “open-like” and closed σ_1_R conformation. In line, we recently observed that H_2_O_2_ and (+)-SKF-10047, but not PRE-084, promote σ_1_R binding to GABARAP (Baeken et al., 2025). An important consideration for the development of novel σ_1_R agonist is whether induction of the σ_1_R–GABARAP interaction is required for their neuroprotective effects, particularly in the context of autophagy modulation. For example, although PRE-084 does not induce σ_1_R–GABARAP interaction in HeLa cells (Baeken et al., 2025), it enhances autophagic flux in both HeLa cells and *C. elegans*, possibly via ULK1 modulation (Christ et al., 2019; Wang et al., 2021). These findings support the idea that σ_1_R agonists can elicit ligand-specific effects, including differential engagement of the σ_1_R–GABARAP axis.

## σ_1_R in GABARAP logistics

A peculiarity concerning σ_1_R and GABARAP is their phylogenetic co-emergence in the *Parahoxozoa*, the earliest metazoan clade with neuronal circuits, suggesting a fundamental role for their interplay in neuronal homeostasis. Intriguingly, choline has been identified as an endogenous σ_1_R agonist (Brailoiu et al., 2019). In neurons, intracellular choline levels rise under oxidative or metabolic stress (e.g., phosphatidylcholine breakdown, hypoxia, or low acetyl-CoA), conditions that typically demand autophagy activation to degrade damaged components or replenish carbon sources. Thus, σ_1_R activation might redirect GABARAP toward autophagic pathways. It remains unclear whether σ_1_R activation sequesters Golgi-resident GABARAP or acts only on newly synthesized protein. The latter could explain σ_1_R’s neuroprotective effects, as it would preserve the Golgi’s GABARAP pool needed for plasma membrane homeostasis. Testing GABARAP–GOLGA2 interactions after σ_1_R activation may resolve this question.

Another group of molecules that modulate this system are steroid hormones: dehydroepiandrosterone (DHEA) acts as a σ_1_R agonist, while progesterone functions as an antagonist (Moriguchi et al., 2011; Johannessen et al., 2011). This aligns with σ_1_R’s evolutionary relationship with ergosterol-synthesizing sterol isomerases in yeast (Hanner et al., 1996). Notably, DHEA also serves as a negative allosteric regulator of GABA_A_ receptors (Genud et al., 2009), suggesting that simultaneous σ_1_R activation and GABARAP rerouting could alter synaptic tone. Conversely, progesterone acts as a positive GABA_A_ receptor modulator (Kapur and Joshi, 2021), effectively mirroring DHEA’s opposing effects on this dual regulatory axis.

**FIGURE 2:**
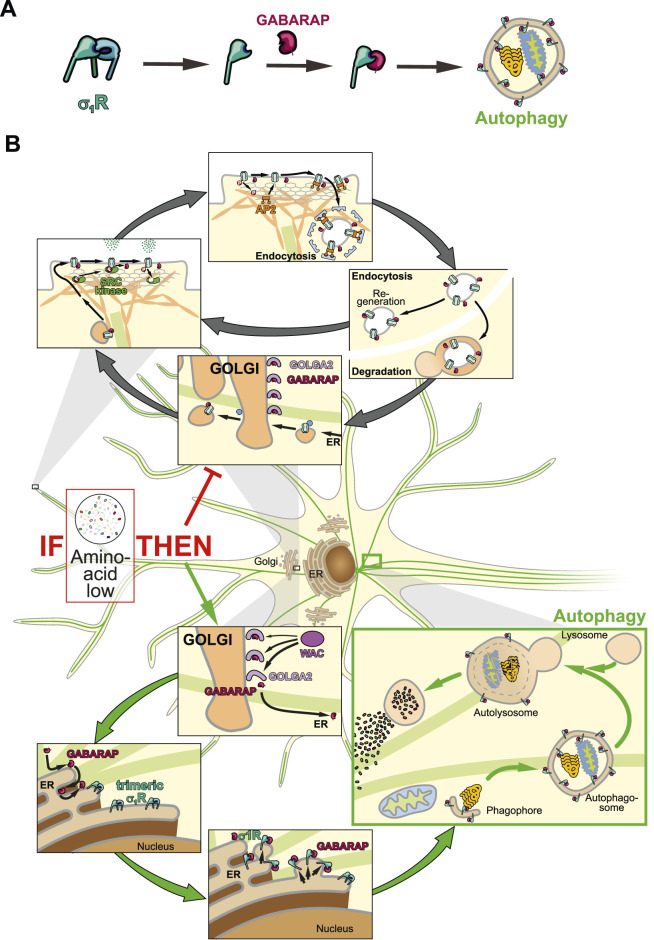
Schematic illustration of neuronal GABARAP logistics. **(A)** Step by step illustration of σ_1_R activation leading to autophagosome-associated GABARAP. **(B)** GABARAP is stored at the Golgi and either mediates receptor transport or integrates into autophagy via σ_1_R.

Interestingly, crystal structures of *Xenopus laevis* σ_1_R in complex with DHEA sulfate (DHEAS) or progesterone demonstrate deeper penetration of the antagonist toward the C-terminal, membrane-associated helix (Fu et al., 2024) (Figure 1C). Here, progesterone probably interacts with Y^203^, while DHEAS engages W^161^. Former interaction might tighten the β-barrel, thereby blocking GABARAP interaction. Notably, DHEA levels rise during fasting (Grasfeder et al., 2009), suggesting σ_1_R activation in extrahepatic tissues could promote autophagy and thereby couple cellular energy responses to systemic metabolic status. In this light, σ_1_R may help precondition peripheral tissues for nutrient scarcity. Consequently, σ_1_R agonists might mimic aspects of the fasting response by shifting GABARAP from anabolic trafficking roles toward catabolic autophagy (Figures 2A,B). Mutational analysis of Y^203^ and W^161^ is needed to confirm their roles in agonist vs. antagonist binding. The relevance of this regulatory axis could further be tested *in vivo* by monitoring autophagy and σ_1_R monomerization under altered hormonal conditions.

In cell culture, GABARAP is rerouted toward autophagy during starvation despite the absence of DHEAS, suggesting either σ_1_R bypass of or activation by alternative ligands. In neurons, Cytochrome P450 46A1 (CYP46A1), which is also upregulated during starvation (Shinohara et al., 2022), generates oxysterols potentially fulfilling this role. The biomolecular foundation model Boltz-2 predicts that 24(S)-hydroxycholesterol, a CYP46A1 product, binds human σ_1_R (Figure 1C), possibly explaining how CYP46A1 enhances autophagy (Nóbrega et al., 2019) and how H_2_O_2_ induces σ_1_R activity via sterol oxidation. Although further tests with oxysterol treatments and monitoring of σ_1_R and autophagy are required, current evidence highlights σ_1_R as a key regulator of GABARAP-mediated cell functions.

## Data Availability

The original contributions presented in the study are included in the article/supplementary material, further inquiries can be directed to the corresponding author.
